# Prediction of Significant Coronary Artery Disease Through Advanced Echocardiography: Role of Non-invasive Myocardial Work

**DOI:** 10.3389/fcvm.2021.719603

**Published:** 2021-08-24

**Authors:** Jolanda Sabatino, Salvatore De Rosa, Isabella Leo, Antonio Strangio, Carmen Spaccarotella, Alberto Polimeni, Sabato Sorrentino, Giovanni Di Salvo, Ciro Indolfi

**Affiliations:** ^1^Division of Cardiology, Department of Medical and Surgical Sciences, “Magna Graecia” University, Catanzaro, Italy; ^2^Cardiovascular Research Center, Magna Graecia University, Catanzaro, Italy; ^3^Department of Women's and Children's Health, University of Padua, Padua, Italy; ^4^Mediterranea Cardiocentro, Naples, Italy

**Keywords:** coronary artery disease, global strain, myocardial work, speckle tracking echocardiography, systolic function, atherosclerosis

## Abstract

**Background:** Non-invasive prediction of critical coronary artery stenosis (CAST) in patients with coronary artery disease (CAD) is challenging. Strain parameters can often capture an impairment of regional longitudinal function; however, they are load dependent. A novel non-invasive method to estimate Myocardial Work (MW) has been recently proposed, showing a strong correlation with invasive work measurements. Our aim was to investigate the ability of non-invasive MW to predict the ischaemic risk area underlying a CAST.

**Methods and Results:** The study population comprises 80 individuals: 50 patients with CAST and 30 controls (CTRL). Echocardiography recordings were obtained before coronary angiography to measure global longitudinal strain (GLS), Myocardial Work Index (MWI), Myocardial Constructive Work (MCW), Myocardial Wasted work (MWW), Myocardial Work Efficiency (MWE). Global MWI (*p* = 0.048), MWE (*p* < 0.001), and MCW (*p* = 0.048) at baseline were significantly reduced in patients with CAST compared to controls (*p* < 0.05). Regional MWE within the myocardial segments underlying the CAST, but not LS, was significantly reduced compared to non-target segments (*p* < 0.001). At ROC analysis, the diagnostic performance to predict CAST for regional MWE (AUC = 0.920, *p* < 0.001) was higher compared to both regional post-systolic shortening index (PSI) (AUC = 0.600, *p* = 0.129) and regional LS (AUC = 0.546, *p* = 0.469).

**Conclusions:** Non-invasive estimation of MW work indices is able to predict a CAST before invasive angiography.

## Introduction

Reliable identification of inducible ischemia in patients with suspected coronary artery disease (CAD) is clinically crucial to their management (1, 2). However, non-invasive prediction of critical coronary artery stenosis (CAST) is challenging, leading to unnecessary invasive angiographies (3, 4).

Despite most patients with significant coronary stenosis have preserved left ventricular (LV) ejection fraction (EF) at rest, regional wall motion abnormalities (WMAs) may be found, but they are often too subtle to be captured.

Current non-invasive risk stratification algorithms are time consuming (2), not sufficiently accurate to prevent redundant invasive angiographies, and mostly not indicated in the emergency setting (5, 6).

Two-dimensional speckle tracking echocardiography (2D-STE) has demonstrated in recent studies to be capable of identify an impairment of longitudinal function downward of CAST (7–13). Furthermore, post-systolic shortening is a sensitive marker of inducible ischemia (14, 15). However, as strain parameters are load dependent, they might not be able to assess systolic function accurately (16, 17).

Myocardial Work Index (MWI), a non-invasive method recently proposed to assess myocardial work using longitudinal strain and a standardized LV pressure (LVP) curve (18–20).

Boe et al. (21) demonstrated that regional MWI is a sensitive marker of acute coronary occlusion (ACO). However, their study was conducted on patients with acute myocardial infarction (AMI), and could not assess the ability of regional MWI to recognize CAST in patients with stable CAD. Another study demonstrated in a non-acute setting that non-invasive MW parameters were sensitive marker of myocardial ischemia during induced transient ACO (22).

Aim of this study was to test whether non-invasive global and regional myocardial work indices might be able to identify the myocardial area underlying a CAST (functional ischemic risk area, FRA) to reduce the number of unnecessary coronary angiographies.

## Materials and Methods

### Study Population

All patients undergoing coronary angiography at a single center during the study period were screened for this study. Among them, 50 consecutive patients with one critical coronary artery stenosis (CAST) at coronary angiography were included into the CAST group and 30 consecutive patients with no critical coronary stenoses were included into the control group. The following criteria have been used as inclusion criteria:

- ≥18 years of age;
- Planned coronary angiography;
- Presence of a critical coronary stenosis, diagnosed during coronary angiography;
- Patients gave their consent.

An acute myocardial infarction (AMI) within 30 days, QRS-width of ≥120 ms, severe valvular disease, previous cardiac surgery, extensive comorbidity, or the presence of atrial fibrillation at ECG were exclusion criteria. All study patients underwent echocardiography and invasive coronary angiography. All patients were stable and provided informed consent. The study was approved by the regional ethics committee.

### Study Timeline, Procedures, and Analysis Plan

A complete echocardiography examination along with measurement of non-invasive automatic oscillometric blood pressure (NINV) were obtained before performing coronary angiography.

Aim of the study was the evaluation of the diagnostic performance of STE and non-invasive echocardiography-derived Myocardial Work parameters to predict CAST (≥70%) before the invasive study. To this purpose, baseline measurements of all study patients were assessed and compared to CTRLs. Regional LV performance indices, calculated in the functional ischemic risk area (FRA), downward of CAST, were compared to the remaining (non-ischemic) myocardial segments.

### Echocardiographic Analyses

Two-dimensional (2D) 4-chambers, 3-chambers, and 2-chambers apical views were acquired with a frame rate ≥60 fps, as described elsewhere (23), and transferred to a dedicated workstation (EchoPAC, GE Healthcare). The echocardiographic recordings were processed using a semi-automatic speckle-based strain software analysis (EchoPAC version 112.99, Research Release, GE Healthcare). LV peak systolic, longitudinal strain (LS), and post-systolic Shortening Index (PSI) were calculated by tracing the LV endocardium's inner border in each view. The EchoPAC software automatically identifies the region of interest (ROI), then approved by the operator supervising the whole process in order to ensure the proper delimitation of the LV wall and the correct assessment of the wall thickness into the analysis. Finally, data of segmental and global LV peak systolic LS and PSI, segmental and global, were processed and analyzed by the software through an 18-segment model. PSI was calculated as previously described by Kukulski et al. [PSI = (peak systolic strain – end-systolic strain)/peak systolic strain] (7).

### Calculation of Non-invasive Myocardial Work

MWI was calculated as the area under the LV pressure-strain curve (GE-Healthcare, Chicago, IL, USA). Moreover, the following additional indices were calculated:

- Myocardial Constructive Work (MCW): positive work performed during systolic shortening plus negative work during lengthening in isovolumic relaxation (IVR);
- Myocardial Wasted Work (MWW): negative work generated during systolic lengthening adding the work performed during LV shortening in IVR;
- Myocardial Work Efficiency (MWE): ratio between constructive work and the sum of constructive and wasted work (0–100%).

Reproducibility was assessed on a subset of randomly selected echocardiograms (*n* = 15). Two operators independently and blindly performed the assessment and one of the two additionally analyzed the same series of echocardiographic exams a second time blindly. Inter- and intra-rater reliability were then assessed using the intraclass correlation coefficient (ICC).

### Evaluation of Regional Function in Patients With Critical Coronary Stenosis

The functional ischemic risk area (FRA) and the non-risk areas (NRA) were selected according to the vascular distribution of the coronary vessels with or without critical coronary stenosis (CAST), as previously described (24). Briefly, the inferior mid- and basal segments imaged in the apical two-chamber view were considered FRA segments in presence of a CCS of the right coronary artery (RCA). In presence of a CAST of the left circumflex coronary artery (LCx), the basal- and mid- lateral segments in the apical four chamber view were considered “at-risk” (25). Both septal mid- and apical segments from the apical four-chamber view, were considered “at-risk” in presence of a CAST of the left anterior descending coronary artery (LAD), as previously described (21, 24, 25).

Empirical cut-off values were set to identify segmental systolic dysfunction for MWI and according to Boe et al. published data (21). The presence of ≥4 adjacent dysfunctional segments assessed by MWI were used to assess the FRA area in order to detect the presence of CAST.

### Data Analysis and Statistics

Continuous values are expressed as mean ± SD. Mean differences between groups were analyzed with ANOVA and the unpaired Student's *t*-test. The Mann Whitney *U*-test was used to compare two groups for non-normally distributed variables. Intra- and inter-rater agreement was assessed using the intraclass correlation coefficient (ICC).

Discrete variables are reported as absolute numbers and percentages of the group. Comparisons between discrete variables were performed using the Chi-squared test.

Receiver operating characteristic (ROC) curve analysis was used to identify optimal cut-off values, using the Youden method. Comparisons between the AUC of different ROC Curves were performed using the DeLong method, as already described (26, 27).

Net reclassification improvement (NRI) index was calculated to quantify the proportion of tests being correctly reclassified with a test with a higher AUC compared to another, as already elsewhere described (28–30).

The statistical analyses were performed using IBM SPSS v.21.1 (IBM corp., Armonk, NY, USA). A two-tailed *P*-value of 0.05 was interpreted as significant.

## Results

### Study Population

Eighty patients undergoing coronary angiography for clinical indication have been enrolled in this study, including fifty patients with coronary artery disease (CAD) and critical coronary stenosis (CAST) documented at invasive coronary angiography and 30 controls (CTRL) with no CAST at coronary angiography. Seven patients from the first group were excluded for poor quality of the echocardiographic images, one patient for ongoing ventricular bigeminy, and one for inadequate tracking. CAST patients were slightly older and more frequently smokers compared to controls. Distribution of other risk factors was balanced between the groups. Patient characteristics, including risk factors, biomarkers, and clinical presentation are listed in Table 1. No significant difference regarding the indication for coronary angiography was observed between the groups (Table 1).

**Table 1:** Baseline patients characteristics.

	**CAST (*n* = 41)**	**CTRL (*n* = 30)**	**Significance (*p*-value)**
Age (years)	67 ± 9	61 ± 8	0.036
Male, *n* (%)	32 (78)	15 (50)	0.603
Smoker, *n* (%)	11 (26)	4 (13)	0.021
Hypercolesterolemia, *n* (%)	30 (73)	18 (60)	0.455
Diabetes mellitus, *n* (%)	11 (26)	9 (6)	0.540
Hypertension, *n* (%)	32 (78)	23 (76)	0.527
Creatinine (mg/dL)	0.89 ± 0.21	0.81 ± 0.17	0.139
Troponin T (mg/dL)	0.04 ± 0.07	0.04 ± 0.14	0.873
LBBB, *n* (%)	0 (0)	0 (0)	–
RBBB, *n* (%)	0 (0)	1 (3)	0.361
LAFB, *n* (%)	3 (7)	2 (7)	0.126
AV block, *n* (%)	0 (0)	0 (0)	–
Angina	24 (59)	15 (50)	0.609
Inducible ischemia	11 (27)	12 (40)	0.184
Coronary CT	3 (7)	3 (10)	0.674
Ventricular arrhythmias	1 (3)	1 (3)	0.777
Target vessel, *n* (%)			
• LAD	25 (60)	N/A	–
• LCx	5 (12)	N/A	–
• RCA	11 (26)	N/A	–

### Blood Pressure During the Study

Mean non-invasive blood pressure (BP) values are reported in Table 2. NINV systolic (SBP) and diastolic (DBP) BP values were comparable in patients with CAST at angiography and controls (SBP: 143.8 ± 17 vs. 136.4 ± 12, *p* = 0.08; DBP: 81.2 ± 9 vs. 80.1 ± 12, *p* = 0.44).

**Table 2:** Echocardiographic data.

	**CAST**	**Controls**	**Significance (*p*-value)**
LVEF (%)	55 ± 11	59 ± 4	0.089
GLS (%)	−16.5 ± 4.1	−21.7 ± 2.6	<0.001
SBP	143.8 ± 17	136.4 ± 12	0.080
DBP	81.2 ± 9	80.1 ± 12	0.440
PSI (%)	4.9 ± 4	0.5 ± 0.4	<0.001
MWI (mmHg%)	1843 ± 540	2070 ± 246	0.048
MWE (%)	92.3 ± 5.1	96.6 ± 1.7	<0.001
MCW (mmHg%)	2112 ± 577	2356 ± 269	0.048
MWW (mmHg%)	131.6 ± 91.5	60.6 ± 39.1	<0.001

### Baseline LV Performance Indices in Patients With Critical Coronary Stenoses

**Figure 1:**
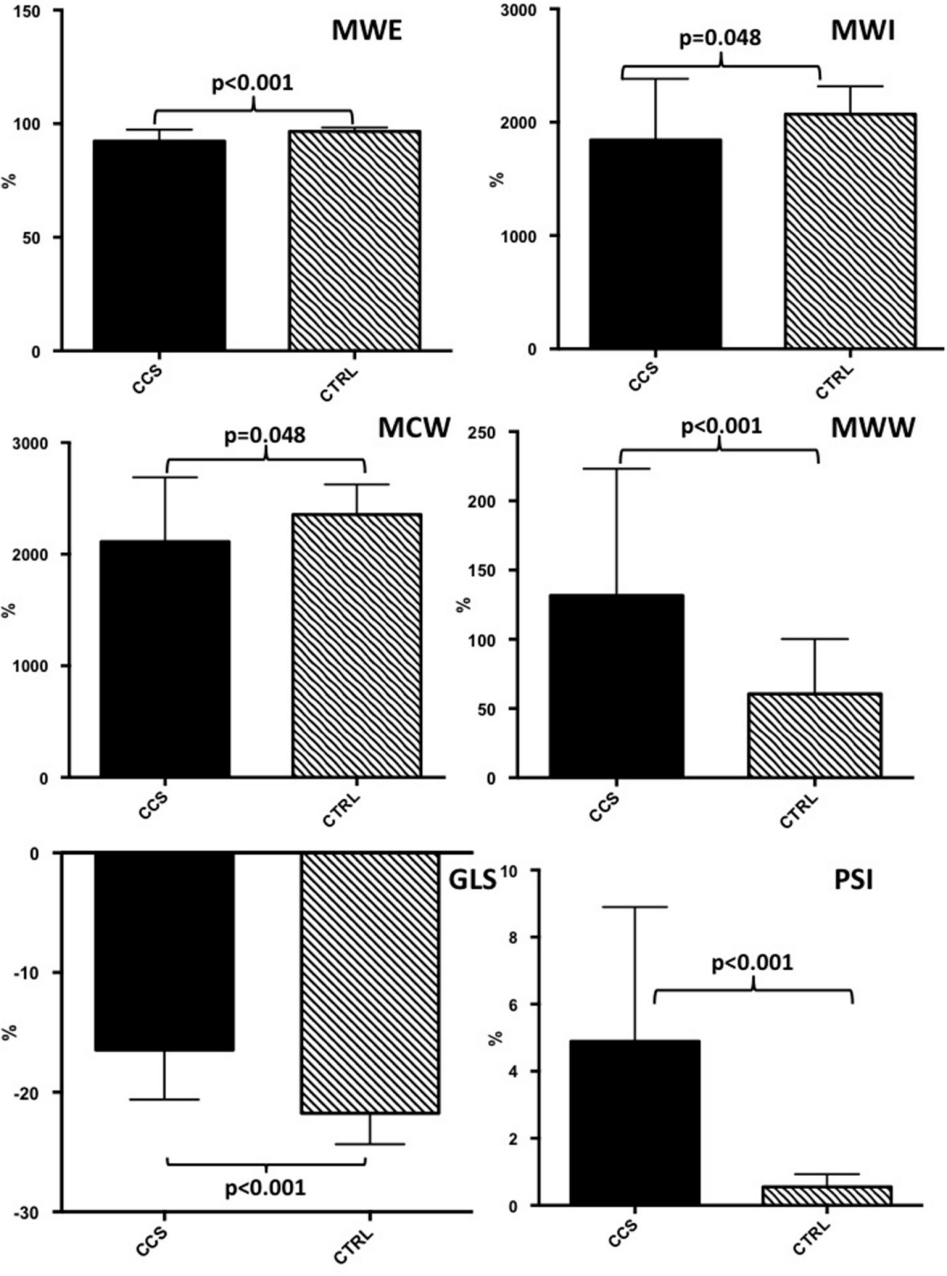
Baseline echocardiographic evaluation of myocardial work indices and global longitudinal strain in patients with critical coronary stenosis compared to healthy controls. CAST, critical coronary stenosis; CTRL, controls; GLS, global longitudinal strain; MWI, myocardial work index; MCW, Myocardial Constructive Work; MWW, Myocardial Wasted Work; MWE, Myocardial Work Efficiency.

Left ventricle Ejection Fraction (LVEF) was within normal range at baseline, without significant difference between the groups (*p* = 0.089). No regional wall motion abnormalities were present at rest. Baseline global MWE (92.3 ± 5.1%) was significantly reduced in patients with CAST compared to controls (96.6 ± 1.7%, *p* < 0.001; Figure 1). Similarly, baseline global MWI (1,843 ± 540 mmHg% vs. 2,070 ± 246 mmHg%, *p* = 0.048), MCW (2,112 ± 577 mmHg% vs. 2,356 ± 269 mmHg%, *p* = 0.048), and GLS (−16.4 ± 4.1% vs. −21.7 ± 2.6%, *P* < 0.001) were significantly reduced in patients with CAST compared to controls (Figure 1).

**Figure 2:**
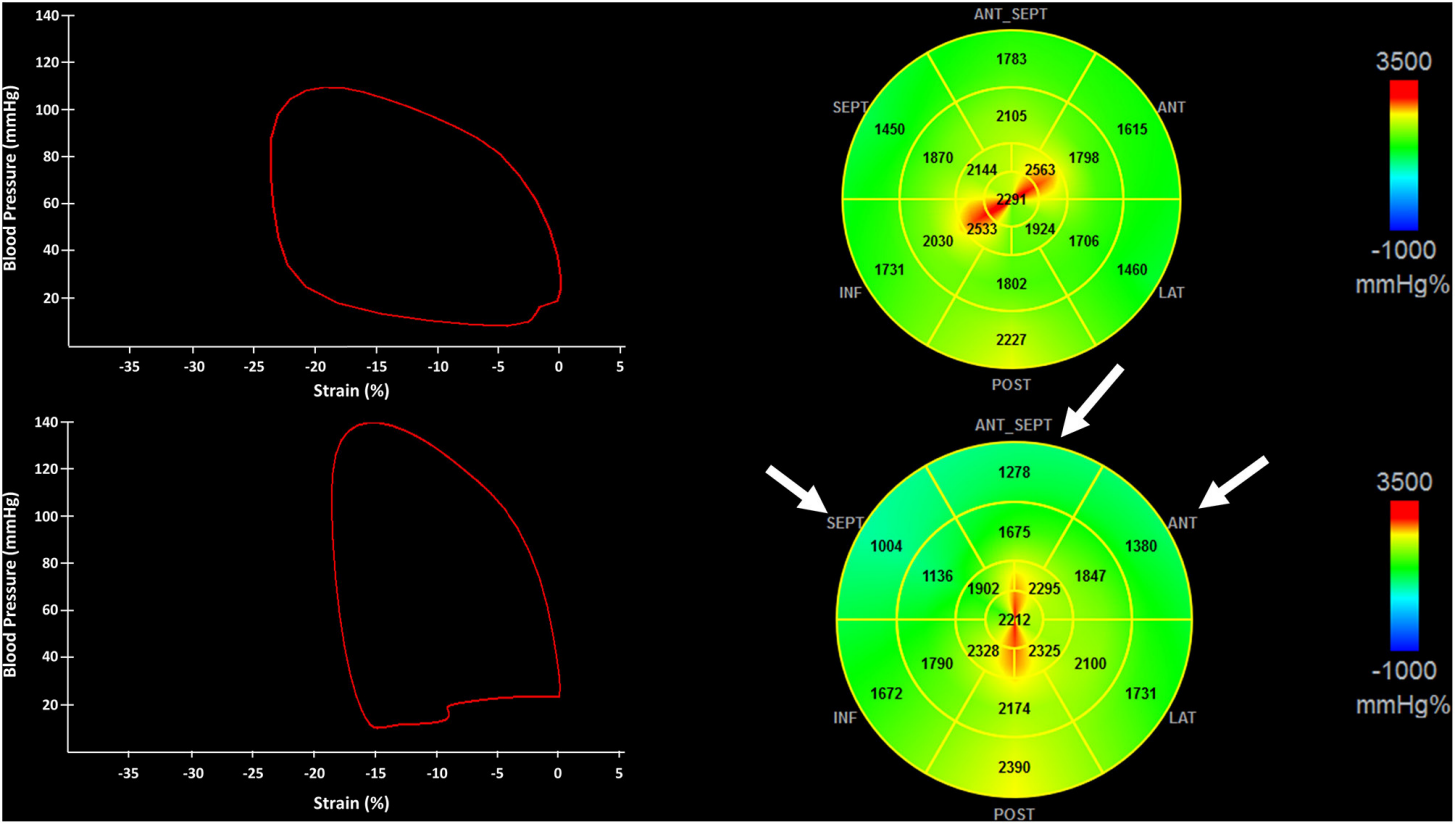
The figure shows pressure-strain loops (Left) and the bullseye of the regional distribution of non-invasive myocardial work index (Right) from a patient with no critical coronary stenosis (CAST) (Upper) and CAST of the left anterior descendent artery (LAD) (Lower). Myocardial segments underlying the LAD stenosis are indicated by white arrows. CAST, critical coronary stenosis; LAD, left anterior descending artery.

Exemplificative pressure-strain curves (left panels) and bullseye views of MWI (right panels) of two patients: one with no coronary stenosis (upper panel) and one with CAST of the Left Anterior Descending (LAD) (lower panel) are shown in Figure 2.

On the contrary, PSI (4.9 ± 4% vs. 0.5 ± 0.4%, *P* < 0.001) and MWW were significantly increased in patients with CAST compared to CTRL (*p* < 0.001). Finally, baseline LVEF in patients with CAST (55 ± 11%) was not different from CTRL (*p* = 0.089). At ROC analysis no difference was found between global MWE and GLS (AUC = 0.850 vs. AUC = 0.850, *p* = 0.816), in terms of diagnostic performance to predict CAST.

**Figure 3:**
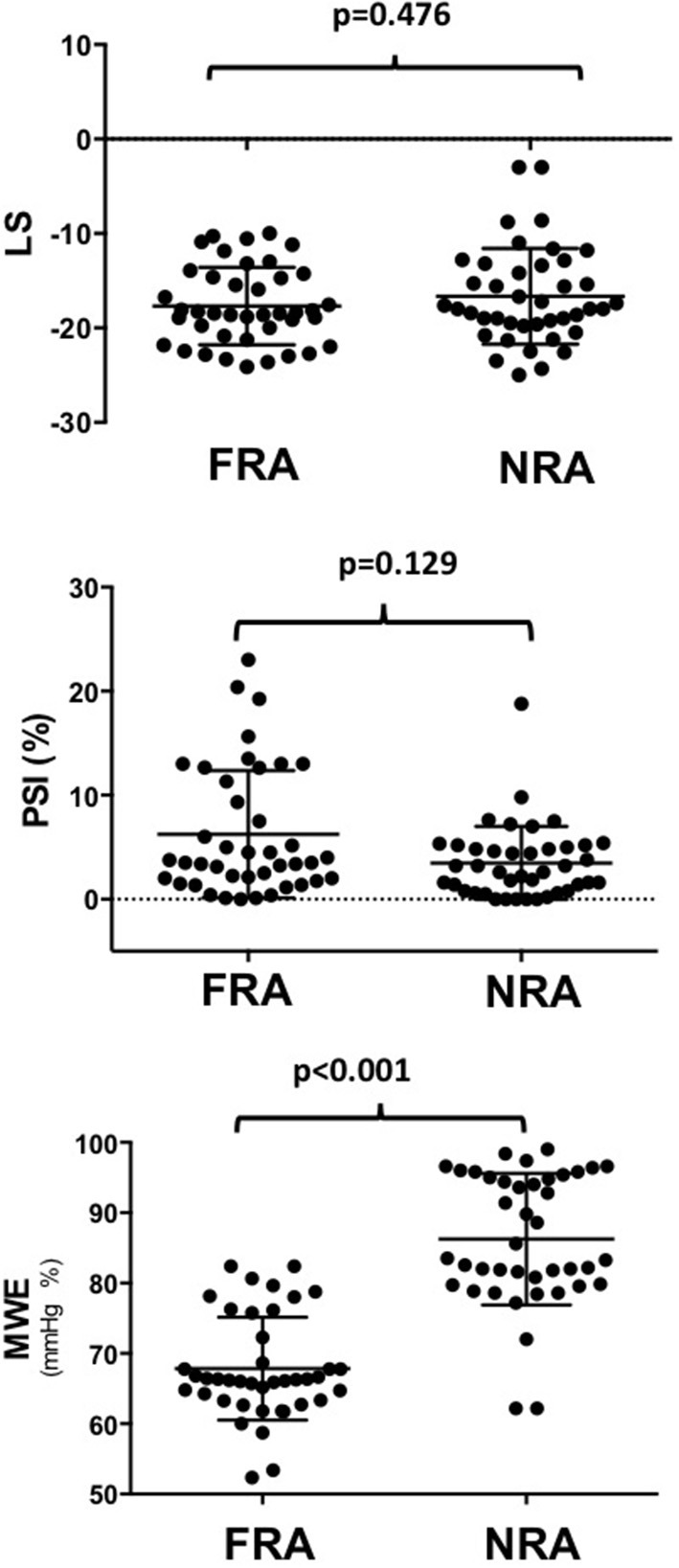
The chart shows that regional MWE, measured within the FRA (underlying the target vessel with CAST), was decreased by 21% (*p* < 0.001) compared to the NRA of the same patients, while no significant change was found for LS (*p* = 0.476), nor for PSI (*p* = 0.129). MWE, Myocardial Work Efficiency; CAST, critical coronary stenosis; LS, Longitudinal Strain; FRA, functional risk area; NRA, non-risk area; PSI, post-systolic shortening index.

**Figure 4:**
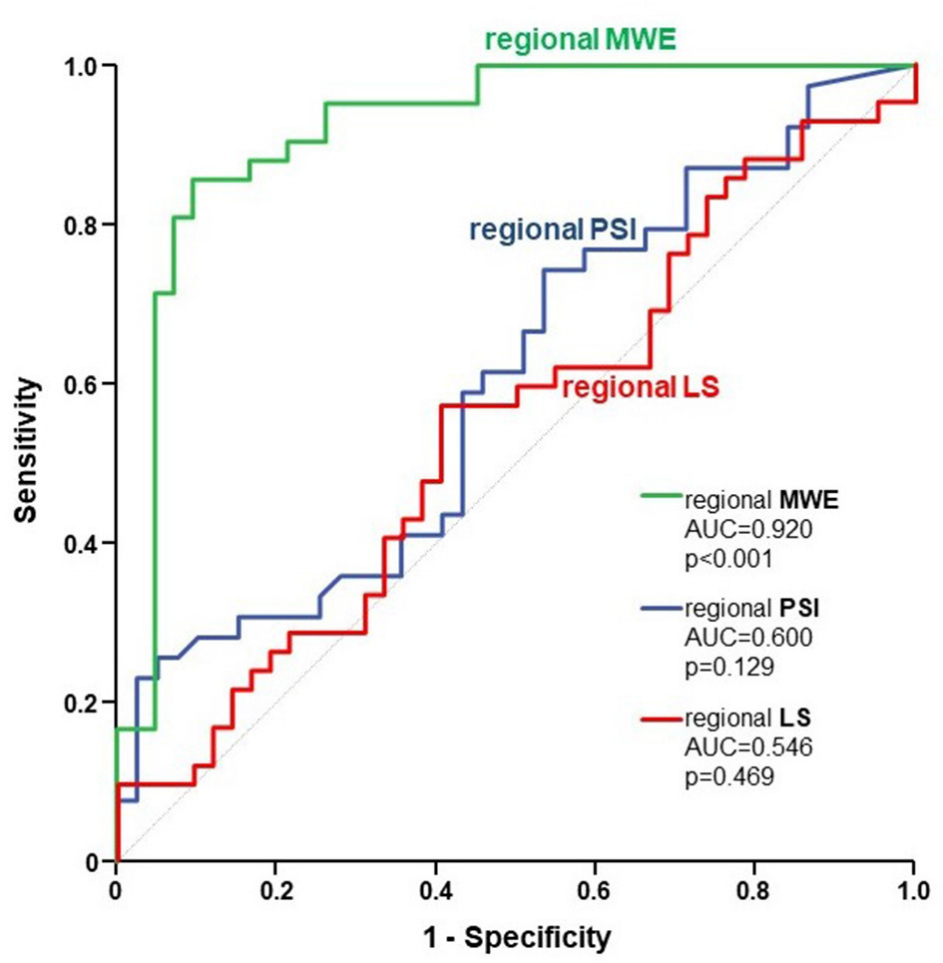
The figure depicts the ROC curves for regional MWE (green), regional PSI (blue), and regional LS (red), reflecting their respective diagnostic performance to predict CAST. ROC, receiver operating characteristic; MWE, Myocardial Work Efficiency; PSI, post-systolic shortening index; LS, Longitudinal Strain; CAST, critical coronary stenosis.

Regional MWE, measured within the FRA (i.e. underlying the target vessel with CAST) was significantly decreased by 21% (*p* < 0.001) compared to NRA of the same patients, while no significant change was found for regional LS (*p* = 0.476). Regional PSI measured within the FRA was not significantly higher than in NRA, although it was numerically higher (5.9 ± 6% vs. 3.5 ± 4%; *p* = 0.129; Figure 3). At ROC analysis, the diagnostic performance to predict CAST for regional MWE (AUC = 0.920, *p* < 0.001) was higher compared to both regional PSI (AUC = 0.600, *p* = 0.129) and regional LS (AUC = 0.546, *p* = 0.469; Figure 4). Accordingly, direct comparison of ROC curves using the method developed by DeLong confirmed a significantly higher AUC for regional MWE compared to both regional PSI (*p* < 0.001) and regional LS (*p* < 0.001; Table 3).

**Table 3:** Diagnostic performance of regional performance indices.

**Index (regional)**	**Cut off (%)**	**Sensitivity (%)**	**Specificity (%)**
LS	−17^*^	45.2	61.9
PSI	6^#^	30.8	84.6
MWE	91^§^	40.5	100
MWE	78^@^	90.5	85.7

* *Clinically validated cutoff for LS (31)*.

# *Clinically validated cutoff for PSI (32)*.

§ *Previously published cutoff for MWE (33)*.

@ *MWE cutoff found in the present study according to the Youden Index method*.

**Figure 5:**
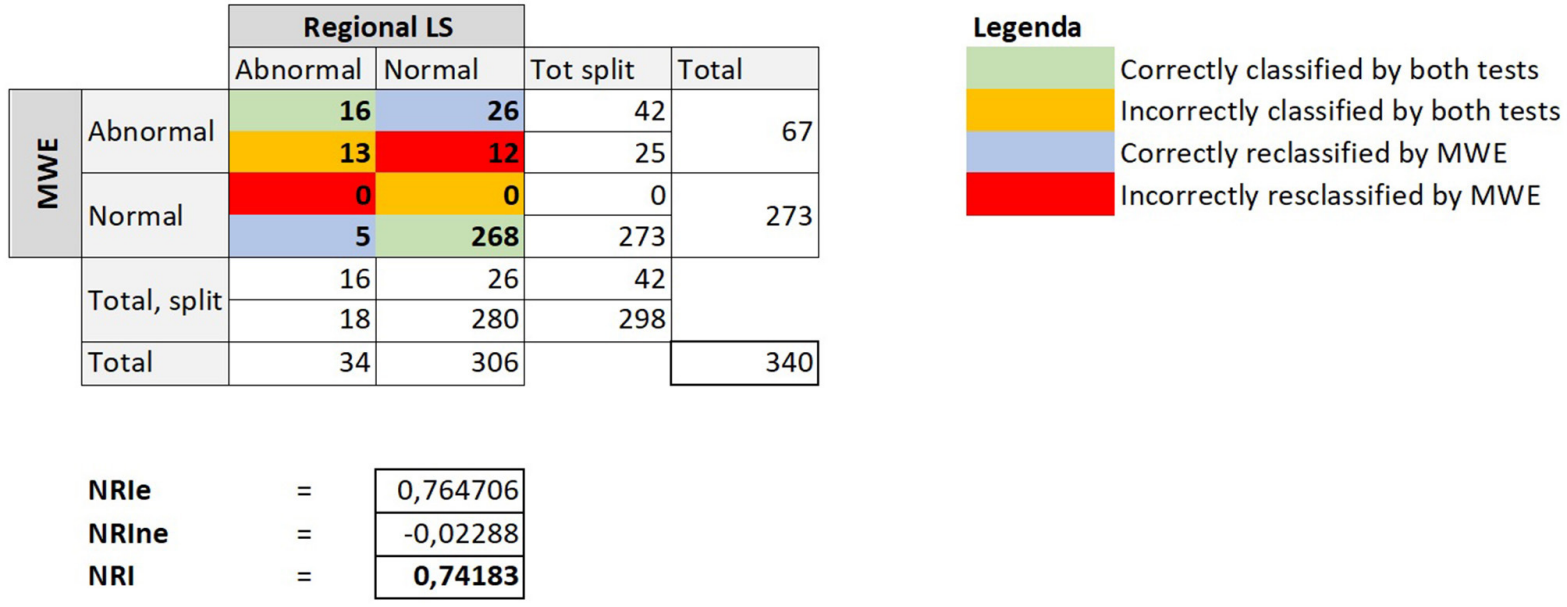
The figure depicts the crosstab comparing the diagnostic classification obtained using regional LS or alternatively regional MWE to identify CAST. The reclassification indices are reported below the tab. MWE, Myocardial Work Efficiency; LS, Longitudinal Strain; CAST, critical coronary stenosis; NRI, net reclassification improvement; NRIe, NRI event; NRIne, NRI no event.

Finally, we compared diagnostic classification by regional indices using Reclassification Statistics, revealing that regional MWE was able to correctly reclassify myocardial segment underlying a CAST in a substantially higher number of segments, as demonstrated by a strong NRI of 0.74 (Figure 5). The amount (segments/total) of FRA and NRA segments is shown in Table 4.

**Table 4:** Amount of reclassified FRA and NRA segments.

	**FRA**	**NRA**
By MWI (segments/total)	0.4	0.3
By GLS (segments/total)	0.3	0.2

### Reproducibility

The assessment of non-invasive myocardial work had an excellent reproducibility. In fact, the intraclass correlation coefficient for intra-rater assessment was excellent for all measured parameters, including GLS (ICC = 0.975; 95% CI: 0.930–0.991), MWI (ICC = 0.967; 95% CI: 0.905–0.988), GWE (ICC = 0.969; 95% CI: 0.911–0.989), GWW (ICC = 0.900; 95% CI: 0.680–0.966), and GCW (ICC = 0.956; 95% CI: 0.876–0.984). Similarly, the intraclass correlation coefficient for inter-rater assessment was excellent for GLS (ICC = 0.875; 95% CI: 0.557–0.960), MWI (ICC = 0.967; 95% CI: 0.905–0.988), GWE (ICC = 0.960; 95% CI: 0.907–0.985), GWW (ICC = 0.962; 95% CI: 0.914–0.986), and GCW (ICC = 0.879; 95% CI: 0.653–0.958). Bland-Altman plots for intra- and inter-rater comparisons are shown in Supplementary Figure 1.

### Correlations

At baseline, global PSI presented a good inverse correlation with global MWE (*R* = −0.646, *p* < 0.001), a weak linear correlation with global MWW (*R* = 0.340, *p* = 0.005), and inverse correlation with global MWI (*R* = −0.286, *p* = 0.018). MWI had an optimal inverse correlation with GLS (*R* = −0.695, *p* < 0.001).

## Discussion

Our results demonstrate that: (1) baseline global MWI, MCW, MWE are significantly worse in patients with CAST compared to CTRLs; (2) regional MWE at baseline is significantly worse in myocardial segments underlying a CAST and might therefore be exploited to identify the myocardial region downward of a CAST before invasive coronary angiography. Regional MWE outperforms both regional PSI and regional LS for this application.

### Myocardial Work and Ventricular Function

Regional LV function is highly dependent on subendocardial blood flow (34). Parameters of longitudinal deformation are dependent by the loading conditions and this could limit their accuracy. Invasively measured Myocardial Work has been historically used as marker of ventricular contractility (35–38). It was later demonstrated that it is feasible to provide similar physiological information as pressure/strain loops (33).

Recently, Russell et al. (18) validated a novel method for non-invasive MW estimation, using of speckle tracking analysis and an estimation of LV pressure from brachial artery cuff pressure. The NORRE sub-study demonstrated a good reproducibility, reporting reference ranges for non-invasive MW (39).

MW indices have been also evaluated by Chan et al. (40) in three different cardiovascular scenarios, such as hypertension, ischaemic, and not-ischaemic dilated cardiomyopathy. Indeed, this group demonstrated a significant increase of MWI in hypertensive patients, despite normal global longitudinal strain values.

All these evidences support the hypothesis that, since myocardial work indices take into account multiple hemodynamic factors, they could represent a valid implementation to the sole strain measurements.

In this context, our results confirm and further extend previous results by Edwards et al. (31). In line with their findings, we found that global MW indices, GLS and PSI at baseline were significantly different in CAD patients as compared to controls. However, unlike them, at ROC analysis we found a similar diagnostic performance of global MW indices and GLS, whereas Edwards et al. (31) reported a modest—although statistically significant—superiority of global MW (AUC = 0.786) compared to GLS (AUC = 0.693). The reasons for this slight discordance are most probably multifactorial. First, the slightly lower global LV function in our study (mean EF 55 vs. 62% in the study by Edwards et al.) might have partially attenuated the impact of regional ischemia on global LV performance indices. On the other hand, the study population included by Edwards et al. presents a lower degree of interindividual heterogeneity which advantages comparisons between the groups, while our population represents a real-World sample and is therefore nearer to actual clinical practice. In addition, Edwards et al. had a large prevalence of multivessel CAD (62.5% among CAD patients), which would suggest larger ischemic areas. The smaller ischemic areas in our all-comers population might have diluted the impact of regional impairment of LV performance on global indices. In this regard, a key novelty of our study compared to the previous by Edwards et al. is the assessment of regionalized LV performance indices. In particular, regional MWE might solve one of the most relevant limitations of the approach proposed by Edwards et al., namely the very low specificity. In fact, in the present study regional MWE had a 100% specificity using the cut off previously proposed by the NORRE Study (32, 39, 41).

### Myocardial Function in Patients With Critical Coronary Stenoses

Regional and global myocardial work non-invasive assessment by LV pressure-segment length loops is a feasible method of evaluate myocardial performance during ischaemia in an experimental setting as recently demonstrated by Boe et al. (21).

CAST are associated with chronic myocardial blood flow reduction, which may cause impairment in regional function within the ischaemic risk area. However, the echocardiographic documentation of the resulting impairment in systolic function is frequently challenging. In fact, its manifestation is not only related to the myocardium contractile state, but it is also sensitive to variations in preload and afterload. Indeed, previous studies have already demonstrated that an elevation in afterload may cause a further decrease in systolic shortening of an ischaemic segment (42).

Boe et al. (21) exemplified how regional contraction may be influenced by load conditions, showing that segments can falsely be interpreted as dysfunctional using strain values alone in case of high systolic blood pressures. In fact, they (21) elegantly demonstrated that MWI reclassified several of those segments as normal, correcting in this way the falsely interpreted impairment in systolic function derived by the strain measurement in patients with a high afterload and no coronary occlusion. In line with those findings, our study complements results by Boe et al. (21) demonstrating for the first time that use of regional MWE is able to identify at baseline CAD patients with CAST before invasive angiography with excellent performance (AUC = 0.92), providing a substantial improvement of specificity compared to global LV performance indices in this setting. The use of regional MWE was superior to both global LV performance parameters than and other regional LV deformation indices. In particular, use of MWE was able to correctly reclassify most patients with a strong NRI of 0.74 compared to regional PSI. Further differences between MWI and strain are related to the hypothesis that peak strain values have restricted ability to reveal myocardial performance. As a matter of fact, peak strain is determined by the afterload concurrently with contraction, while numerically equal peak segmental strain values are not associated to the same level of segmental systolic function in early vs. mid-systole, reflecting the impact exerted by a higher afterload in mid-systole. Our results extend and complement previous findings (7, 8, 43). In our study, global PSI had a direct correlation with global MWW but an inverse correlation with global MWE. This is compatible with the hypothesis that the adjunctive post-systolic LV deformation measured as an increased PSI under myocardial ischemia mostly generates wasted work in the FRA, reducing global LV mechanics efficiency.

### Application of Non-invasive Myocardial Work in Clinical Practice

The findings of this study might have a significant impact on clinical practice. In fact, approximately 60% of coronary angiograms performed for clinical indication find no critical coronary stenoses, according to the National Cardiovascular Data Registry of the American College of Cardiology, exposing a large number of patients to the avoidable risks of an invasive diagnostic procedure (44). In this context, the use of a non-invasive ultrasound-based screening test could be useful to improve the clinical management process of patients with suspected CAD, a currently unmet need in clinical practice. Several diagnostic methods have been tested already for this purpose, with unsatisfactory results. Among the others, the delay of onset of flow mediated dilation (FMD) seems to be the most promising, yet being able to predict the presence of CAST in only 50% of patients with evidence of coronary stenosis above 70% at invasive angiography (45). On the other hand, the Randomized Clinical Trial CONSERVE Trial (clinicaltrial.gov: NCT01810198) and the CE-MARC 2 Trial (clinicaltrial.gov: NCT01664858) recently demonstrated that use of CT angiography of MRI/SPECT could help reducing the rate of non-useful invasive coronary angiograms, although they expose patients to ionizing radiation and/or high magnetic fields.

The results of the present study should be contextualized in the larger picture of clinical practice. In particular, it is important to take into account pre-test probability (PTP), namely the likelihood that the patient has coronary atherosclerosis and thus CAST. This depends in turn on the prevalence of CAD in the study population. Diagnostic testing is most useful when the likelihood is intermediate. The sample size of the present study did not allow stratification based on PTP. Nevertheless, most patients were in the intermediate PTP range. It is paramount to assess PTP before taking any clinical decision or performing a diagnostic test. I fact, a low PTP should suggest a conservative approach with no further diagnostic testing, as these patients have an excellent prognosis without clinical action. On the other hand, patients with high or very high PTP shouldn't undergo intermediate diagnostic tests, as a false negative result might eventually avoid the appropriate clinical pathway (46). Of course, PTP also impacts the results of the present study. Consequently, these results might not apply to populations with substantially different PTP.

Our finding that regional MWE was significantly impaired in the FRA has potential clinical implications, as it could be of help to guide percutaneous treatment in case of multivessel disease with multiple intermediate stenoses.

### Limitations

Echocardiographic images in 7 patients (16%) were not analysable due to poor image quality. These results are in agreement with recent studies (33). However, this could limit the applicability of our findings. On the other hand, the contrast agent used during the study might improve feasibility. Although the results coherently point in the same direction, the limited sample size does not allow to draw a definite conclusion on this topic, neither they can be generalized to different cohorts. The present results should be regarded as preliminary and hypothesis-generating and need to be validated in larger studies before they can be applicable.

## Conclusions

Global and regional assessment of non-invasive Myocardial Work Indices under basal conditions is potentially able to identify myocardial segments underlying CAST. Large prospective studies will be needed to validate our findings and to confirm whether they might be helpful to improve clinical stratification of patients with suspected coronary stenosis.

## Data Availability Statement

The raw data supporting the conclusions of this article will be made available by the authors, without undue reservation.

## Ethics Statement

The studies involving human participants were reviewed and approved by Magna Graecia University Catanzaro. The patients/participants provided their written informed consent to participate in this study.

## Author Contributions

JS and SD: conceptualization. SD and CI: methodology. AS, IL, AP, and SS: formal analysis. JS and AP: writing—original draft preparation. CI, GD, JS, and SD: writing—review and editing. CS, AP, and SS: visualization. CI: supervision. All authors contributed to the article and approved the submitted version.

## Conflict of Interest

The authors declare that the research was conducted in the absence of any commercial or financial relationships that could be construed as a potential conflict of interest.

## Publisher's Note

All claims expressed in this article are solely those of the authors and do not necessarily represent those of their affiliated organizations, or those of the publisher, the editors and the reviewers. Any product that may be evaluated in this article, or claim that may be made by its manufacturer, is not guaranteed or endorsed by the publisher.
