# Supplementary figures and images for: Prediction of Significant Coronary Artery Disease Through Advanced Echocardiography: Role of Non-invasive Myocardial Work
Source: Front Cardiovasc Med. 2021 Aug 24;8:719603. doi: 10.3389/fcvm.2021.719603 (PMC8421730; doi:10.3389/fcvm.2021.719603)

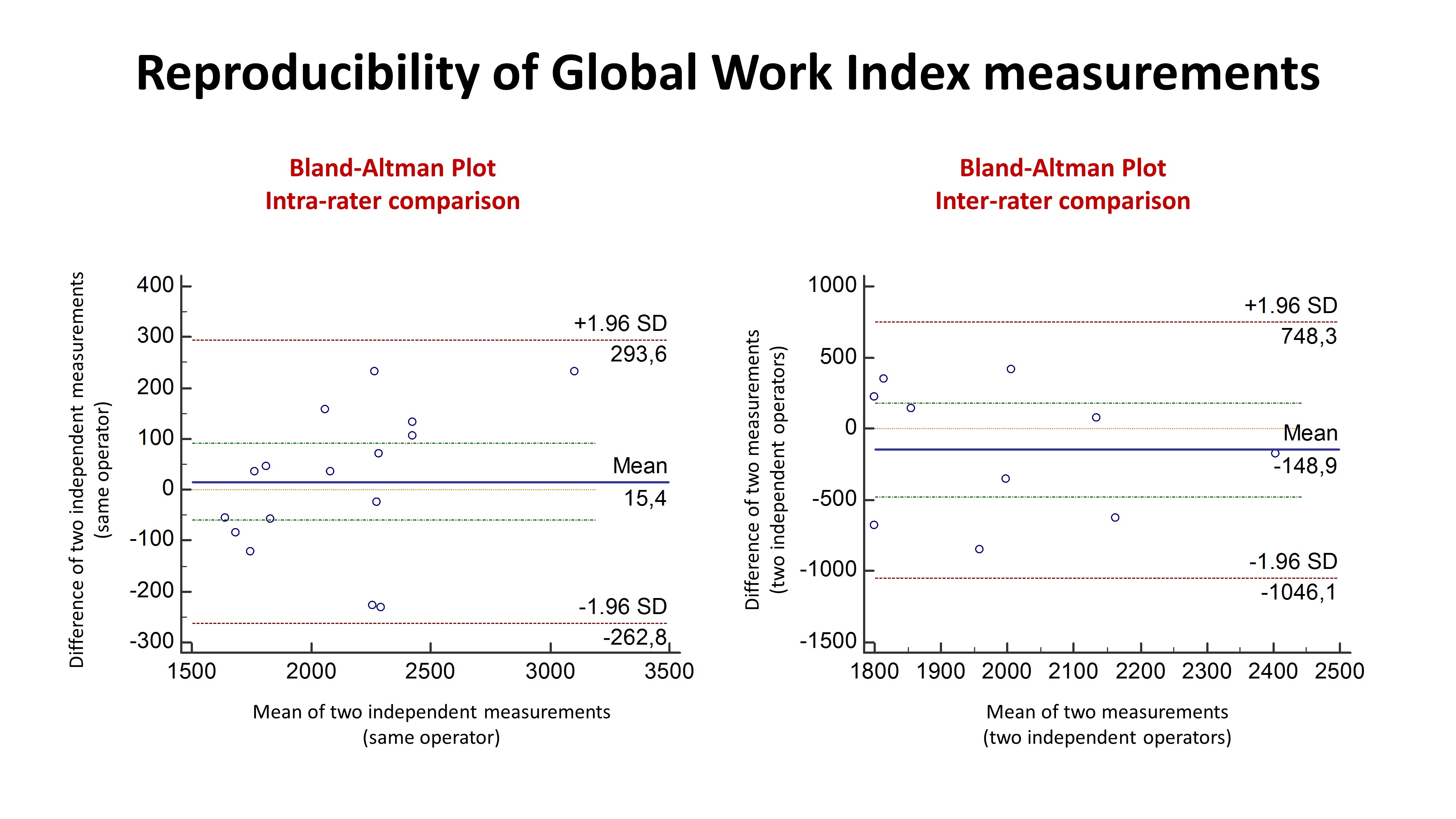

Supplement: Supplementary file 1 [file Image_1.JPEG]
